# Enhancing wound healing dressing development through interdisciplinary collaboration

**DOI:** 10.1002/jbm.b.34861

**Published:** 2021-05-17

**Authors:** Briauna Hawthorne, J. Kai Simmons, Braden Stuart, Robert Tung, David S. Zamierowski, Adam J. Mellott

**Affiliations:** ^1^ Department of Plastic Surgery University of Kansas Medical Center Kansas City Kansas USA

**Keywords:** biomedical engineering, interdisciplinary teams, negative pressure wound therapy, wound dressings, wound healing

## Abstract

The process of wound healing includes four phases: Hemostasis, inflammation, proliferation, and remodeling. Many wound dressings and technologies have been developed to enhance the body's ability to close wounds and restore the function of damaged tissues. Several advancements in wound healing technology have resulted from innovative experiments by individual scientists or physicians working independently. The interplay between the medical and scientific research fields is vital to translating new discoveries in the lab to treatments at the bedside. Tracing the history of wound dressing development reveals that there is an opportunity for deeper collaboration between multiple disciplines to accelerate the advancement of novel wound healing technologies. In this review, we explore the different types of wound dressings and biomaterials used to treat wounds, and we investigate the role of multidisciplinary collaboration in the development of various wound management technologies to illustrate the benefit of direct collaboration between physicians and scientists.

## 1 INTRODUCTION

A glossary of terms found in the introduction is provided in Table 1.

**TABLE 1:** Glossary of terms found in introduction

Term	Description
Basal lamina	Part of the basement membrane that is in contact with the bottom‐most surface of the epithelial and endothelial cells
Epidermal growth factor	Protein that stimulates growth and proliferation of epithelial cells and fibroblasts
Factor VII	Initiating protein of coagulation cascade
Factor XIII	Enzyme that stabilizes fibrin
Fibrinogen	The inactive precursor of fibrin
Fibroblasts	Cells that produce proteins that make up the extracellular matrix such as collagen
Fibronectin	Glycoprotein involved in cell adhesion
Glycoprotein Ib/IX/V	Platelet adhesion receptor involved in hemostasis
IL‐1β	Interleukin‐1beta Proinflammatory cytokine that can mediate fever
IL‐6	Interleukin‐6 Proinflammatory cytokine that stimulates acute phase protein production by the liver and induces inflammation
IL‐8	Interleukin‐8 Chemoattractant for granulocytes, especially neutrophils
Interferon‐γ	Cytokine that activates macrophages, induces helper T cell differentiation (T_H_1), and induces IgG antibody production
Keratinocyte growth factor 2	Protein that stimulates growth of epidermal keratinocytes
Keratinocytes	Cells that make up a majority of the epidermis and produce keratin and the critical stratum lucidum layer
Laminin 5 β‐3	Protein involved in adhesion of keratinocytes of the epidermis to the dermal skin layer
Leukocytes	White blood cells
Macrophages	A type of larger monocyte of the innate immune response that is involved in phagocytosis
Monocyte chemoattractant protein‐1	Proinflammatory protein that attract monocytes
Myofibroblasts	Fibroblasts capable of contraction
Natural killer cell	A type of white blood cell of the innate immune response. This type of lymphocyte plays a major role in the direct early host rejection of both tumors and virally infected cells.
Neutrophil	Polymorphonuclear granulocytes abundant in acute phase of inflammation.
Phagocytize	Engulfment or “eating” of cells and foreign material
Phosphatidylserine	Phospholipid located in cell membrane
Platelet derived growth factor	Glycoprotein produced by platelets and activated macrophages that acts as a chemoattractant for neutrophils and can induce cell division in mesenchymal cell types
T cells	A type of white blood cell seen in the adaptive immunity responsible for cell mediated immunity. T cells, along with B cells, are the two primary types of lymphocytes that determine the specificity of immune response to antigens.
TGF‐β	Transforming growth factor‐beta Cytokine that stimulates proliferation of epithelial cells and inhibits inflammation
Thrombin	Protease that cleaves inactive fibrinogen to fibrin in coagulation cascade
TNF‐α	Tumor necrosis factor‐alpha Proinflammatory cytokine
Transforming growth factor beta	Inhibits function of immune cells such as T cells, B cells, and monocytes/macrophages^173^
Type III collagen	Collagen predominantly found in skin, blood vessels, and granulation tissue
VEGF	Vascular endothelial growth factor A signaling protein that stimulates vasculogenesis and angiogenesis
VonWillebrand Factor	Glycoprotein produced by endothelial cells that stimulates platelet adhesion and aggregation

Abbreviations: TGF, transforming growth factor; TNF‐α, tumor necrosis factor alpha; VEGF, vascular endothelial growth factor.

### 1.1 Overview of wound healing

Wound healing involves four phases: Hemostasis, inflammation, proliferation, and remodeling.^1^ Wound healing begins with transient vasoconstriction of injured vessels.^2^ In the initial phase, damage to the skin exposes the subendothelial collagen and tissue factor, leading to platelet aggregation.^3^ In this process, Von Willebrand factor (vWF) binds to both the subendothelial collagen and the platelet receptor glycoprotein Ib/IX/V.^4^ This adhesion, as well as thrombin generated by tissue factor, results in platelet activation, degranulation, and conformational change.^5^ Conformational change in glycoprotein IIB/IIIA allows the binding of fibrinogen, a crucial step in platelet aggregation and formation of the platelet plug. Tissue factor and phosphatidylserine, located on the surface of platelets and endothelial cells, form a complex with circulating activated Factor VII, leading to activation of a coagulation cascade which results in the cleavage of fibrinogen to fibrin.^5^ Fibrin activates Factor XIII, which acts to crosslink the fibrin monomers. The final result is a strong blood clot.^6^

The hemostasis process of wound healing leads to the inflammatory process in multiple ways. Platelets can activate the complement cascade, leading to inflammation and the formation of the membrane attack complex (MAC).^7^ Activated complement proteins C3a and C5a cause the degranulation of mast cells, which releases factors, such as histamine, heparin, and proteases.^8^ Mast cells express the enzymes cyclooxygenase (COX) 1 and 2, which generate prostaglandins from the arachidonic acid precursor.^8^ The histamine and prostaglandins that are released as a result of mast cell activation lead to increased vascular permeability^9^ causing the sign localized swelling,^10^ which can be visualized. In addition to increasing vascular permeability, prostaglandins sensitize pain receptors,^9^ act as a neutrophilic chemoattractant, and cause vasodilation.^11^ Prostaglandin induced receptor sensitization leads to pain,^11^ while vasodilation leads to redness and heat^10^; all of which can be appreciated by the patient.

Platelet aggregation leads to the release of neutrophil chemotactic factors such as platelet derived growth factor (PDGF) and transforming growth factor (TGF) beta.^12^ Neutrophils migrate to the site of injury and become trapped in the platelet plug.^12^ There, the neutrophils phagocytize dead tissue and release reactive oxygen species that create an unfavorable environment for bacteria.^12^ An excellent review written by Ellis et al^13^ details the inflammatory phase of wound healing. During the inflammatory phase, the neutrophils express cytokines that recruit additional leukocytes (neutrophils, T cells, and macrophages) to the site of injury, such as tumor necrosis factor alpha (TNF‐α), interleukin (IL) 1β, IL‐6, IL‐8, monocyte chemoattractant protein −1 (MCP‐1).^13^ A study by Theilgaard‐Mönch et al^14^ found that neutrophils upregulate factors that promote wound healing by stimulating angiogenesis (e.g., vascular endothelial growth factor [VEGF] and MCP‐1), stimulating the proliferation of fibroblasts and keratinocytes (e.g., IL‐8, IL‐1β, and MCP‐1), and promoting the adhesion of keratinocytes to the dermal layer (laminin 5β3). Recruited macrophages are activated into the proinflammatory M1 phenotype by damage associated molecular patterns (DAMPs), pathogen associated molecular patterns (PAMPs), and interferon‐gamma (IFN‐γ).^13^ The proinflammatory macrophages phagocytize microbes and cellular debris as well as secrete proinflammatory cytokines and chemokines that increase the number of natural killer cells, helper T cells, and macrophages at the site of injury.^13^ Some macrophages will differentiate into anti‐inflammatory and pro‐regenerative M2 macrophages.^15^ The M2 subset of macrophages assist in wound healing by stimulating fibroblasts, stimulating angiogenesis (e.g., PDGF and VEGF), producing collagen precursors, producing factors that attenuate inflammation (e.g., IL‐10 and TGF‐β), and producing of metalloproteinases (MMPs).^15^

The proliferative phase consists of the epithelization, revascularization, and formation of granulation tissue.^16^, ^17^ Epithelial cells located at the wound edges, upon stimulation by the epidermal growth factor (EGF) and TGF‐α produced by the platelets, begin to proliferate. Stimulated fibroblasts secrete factors, such as keratinocyte growth factor (KGF)‐2, which induce keratinocyte migration.^16^ Keratinocytes migrate along the fibrin blood clot by lamellipodial crawling, until they begin to contact each other.^17^ Growth factors bind to endothelial cells and set off a signaling cascade that results in secretion of proteolytic enzymes that break down the basal lamina, allowing the endothelial cells to proliferate and migrate into the wound to form blood vessels.^17^ Fibroblasts initially synthesize a provisional matrix consisting of collagen, fibronectin, and other substances. In the final step of the proliferation phase, granulation tissue replaces the provisional matrix.^17^ Granulation tissue mainly consists of fibroblasts, new blood vessels, and type III collagen.^18^ Some fibroblasts will differentiate into myofibroblasts, which contract to bring the wound edges closer together.^18^

The remodeling phase (also referred to as the maturation phase) is the longest phase of wound healing and is dependent on a delicate balance between synthesis and degradation.^18^ The type III collagen is lysed by collagenases and subsequently denatured and digested by various proteases.^19^ The type III collagen is replaced by type I collagen, which orients itself in an organized manner that gives it maximal strength against stress forces. Fibroblasts begin to decrease in number, resulting in a relatively acellular scar.^20^ Reduced biological activity leads to apoptosis of vascular tissue,^19^ which can be visualized clinically as a reduction in redness.^19^, ^20^

### 1.2 Intro to wound dressings

Throughout history, many innovations have been made in order to enhance wound healing. As far back as about 2000 BC, there are accounts of Sumerians utilizing poultice‐like materials to cover wounds. In 1550 BC, an Egyptian medical papyrus, currently known as Ebers Papyrus, mentions the use of honey, lint, and grease in wound care, which respectively have antimicrobial, absorbent, and barrier properties that are currently considered essential in wound therapy.^21^ Much later, a Greek physician known as Galen of Pergamum, emphasized the importance of keeping the wound moist.^21^ Prior to the late 1800s, dressings were prepared from unsterilized materials that could be reused, such as old linens, rags, and rope.^22^ In the late 1800s, Gamgee of Birmingham had the idea to combine absorbent cotton with compressing gauze in an aseptic manner for use in wound care.^22^ The concept of keeping the wound moist was not scientifically proven until 1962 when George D. Winter published research that showed epithelization of wounds occurs more rapidly in a moist environment, as opposed to wounds that are exposed to air.^21^, ^23^

According to Weir and C. Tod Brindle, two nurse clinicians, in their chapter on wound dressings,^24^ the ideal dressing should provide a moist environment, remove excess exudate, be able to provide antimicrobial properties if needed, facilitate autolytic debridement, prevent contamination by bacteria, be non‐allergenic, minimize pain, be compatible with support needs, be easily applied and removed, thermally insulate the wound, and be cost effective. If, according to our hypothesis, we also look at the recommendations for an ideal dressing by a research scientist, in this case G.D. Winter, father of moist wound healing,^25^ we would add to this list the qualities of “prevents dehydration and scab formation, is permeable to oxygen, is sterilizable–supplies mechanical protection to the wound–is non‐toxic–conforms to anatomical contours, resists tearing, resists soiling, is not inflammable, has constant properties in a range of temperatures and humidities encountered in use, has long shelf life, has small bulk, is compatible with medicaments…” This approach exemplifies the benefits of combining the work of clinician and scientist, even if after the fact rather than contemporaneously. The basic categories for the wet‐to‐dry wound dressings currently being used include contact layer dressings, film dressings, hydrogels, hydrocolloids, foam dressings, alginates, and antimicrobial dressings.^24^, ^26^ These dressings will be further elaborated in the subsequent section. This review article examines wound dressings and the biomaterials used for them, how the wound dressings are used, and the makeup of the research teams involved in creating the dressings and how that may play a role in the translation into clinical practice.

## 2 TYPES OF WOUND DRESSING

There are a variety of different types of wound dressings available. Each wound dressing has unique properties that can be exploited to fit the needs of a patient. Furthermore, wound dressings can be combined to enhance wound healing. The major types of occlusive wound dressings are described below. Table 2 summarizes the advantages and disadvantages of the following wound dressings.

**TABLE 2:** Summary of advantages and disadvantages of several wound dressings

	Advantages	Disadvantages
Contact layer	Low adherence^24^ Used with secondary dressing^24,26^	May adhere to wound bed when dry^29^ Permeable to bacteria^27^
Semipermeable film	Permeable to gases^27^ Effects growth of anaerobic bacteria^24^ Facilitates autolytic debridement^24^ Impermeable to bacteria^27^ Can be used with secondary dressing Maintains moist wound environment	Nonabsorbent Traps underlying exudate^24^ If unchanged, aids bacterial overgrowth in moist environment^25^
Hydrocolloid	Maintains moist wound environment by gelling with exudate^27^ Facilitates autolytic debridement^27^	Forms a thick, yellow malodorous gel^36^
Hydrogel	Maintains moist wound environment^28,36^ Cooling effect when applied^28,36^ Used with secondary dressing^36^ Multiple forms are available^36^	Low absorptive capacity^28^ Exudate accumulation can lead to wound maceration and bacterial proliferation^34^
Foam	Absorbent^50^ Different forms are available^50^ Protects wound from trauma^26^ Provides thermal insultation^26^ Can be combined with NPWT^50^	Risk of drying in wounds with low exudate^28^
NPWT	Brings wound edges together^57^ Stimulates fibroblasts^57^ Stimulates granulation, particularly angiogenesis^57^ Stimulates epithelial proliferation when used with liner (Prevena™)^57^	Various contraindications^59^
Antimicrobial dressings	Prevents wound contamination^54^	

Abbreviation: NPWT, negative pressure wound therapy.

### 2.1 Contact layer dressings

**FIGURE 1:**
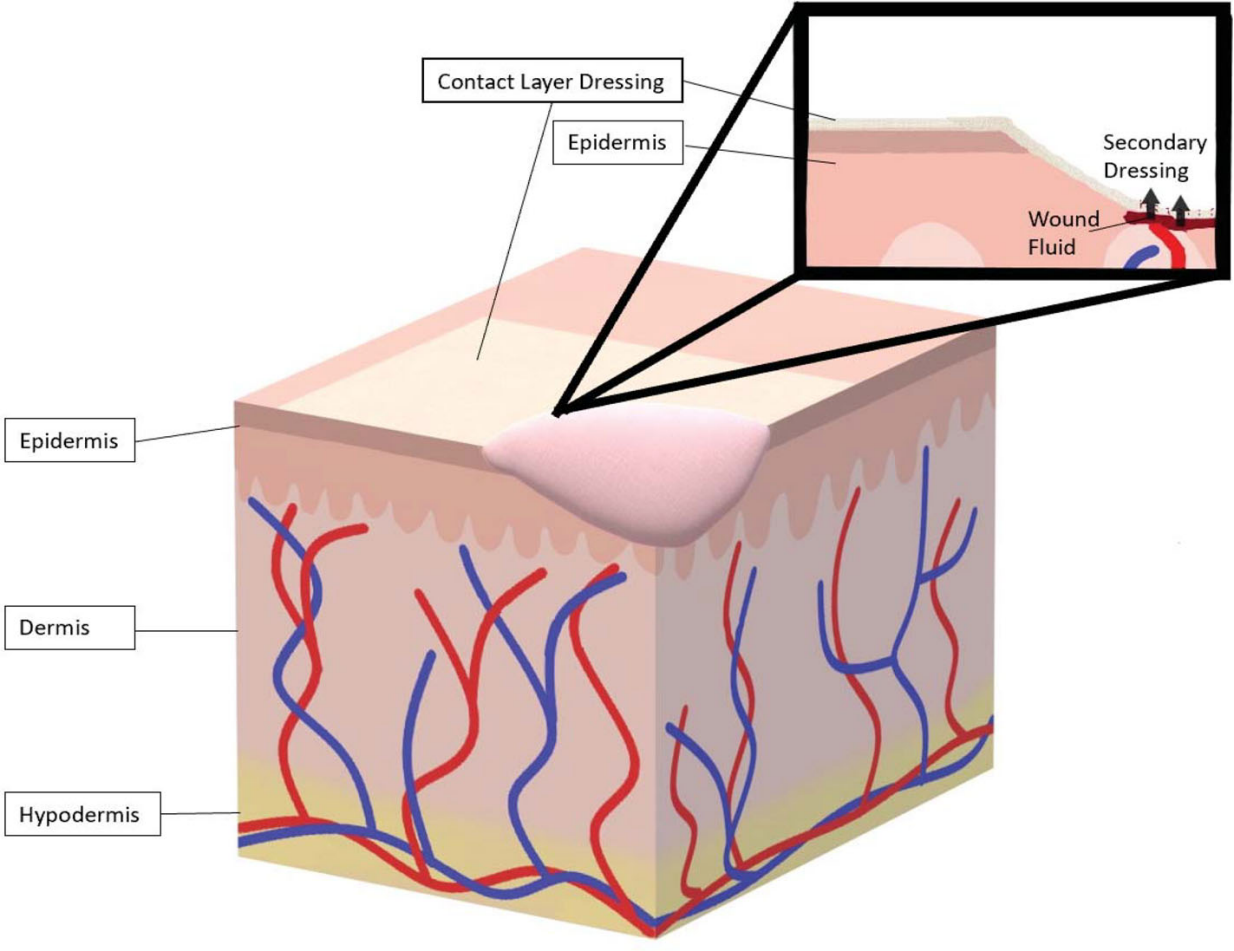
Contact layers. Contact layer dressings provide a cover over of the wound that allows wound fluid such as blood and exudate to flow through to a secondary dressing

Contact layer wound dressings are single layer dressings that are in direct contact with the wound surface. Contact layer wound dressings typically have low adherence with the wound bed thus preventing trauma to the wound bed upon removal.^24^ They function to allow exudate to pass through to a secondary wound dressing^24^, ^26^ and can be used in conjunction with other wound therapies.^24^ Some contact layer dressings, such as gauze impregnated with paraffin, may trap water vapor and lead to maceration, a condition in which exposure to moisture causes the surrounding skin to breakdown.^27^, ^28^ If contact dressings such as gauze impregnated with paraffin and tulle dry out, they may adhere to the wound bed, leading to trauma and pain upon removal of the dressing.^27^, ^29^ Because of these problems, gauze traditionally has attempted to control these factors by the closeness of its weave and the addition of other moisturizing agents. Gauze can be described as having a fine or coarse cotton mesh, depending upon the thread count per inch.^30^ Gauze with a thread count of 41–47, referred to as “fine mesh gauze,”^31^ has been empirically determined to prevent granulation ingrowth through the contact layer, thus enhancing epithelial growth beneath it. When coated with Vaseline™ (Vaseline™ Petrolatum Gauze), or petrolatum, it allows moisture vapor to escape preventing maceration but retains enough moisture to prevent drying. When coated with Bismuth (Xeroform Gauze™) it has the same moisture properties but adds some antimicrobial effect. These two dressings because of their petrolatum base, adhere less to the open wound than other contact layers. Even though they never received the label, these dressings appear to represent the first example of “composite dressings” or, in G.D. Winter's terms, dressings composed of more than one component each with different individual properties. In addition, “Scarlet Red” (a blend of 5% *o*‐tollazo‐*o*‐tolyl‐azo‐B‐naphthol, lanolin, white petrolatum and olive oil^32^) added to fine mesh gauze forms a composite contact layer dressing that deliberately seeks to escarify or scab the wound surface. Finally, other materials besides gauze are used as contact layers. Historically the most important perhaps is rayon (Owen's rayon^33^). This extremely tight weave allows no cellular penetration, but wound exudate quickly incorporates the layer and it becomes part of the more quickly forming, thinner scab when this dressing is used. Contact layer dressings are permeable to bacteria.^27^ Figure 1 illustrates the usage of a contact layer dressing on a wound. Interprofessional cooperation on the front end and review afterward would help problems like the fact that the above information on contact layer dressings does not appear in any one published description.

### 2.2 Semipermeable films

**FIGURE 2:**
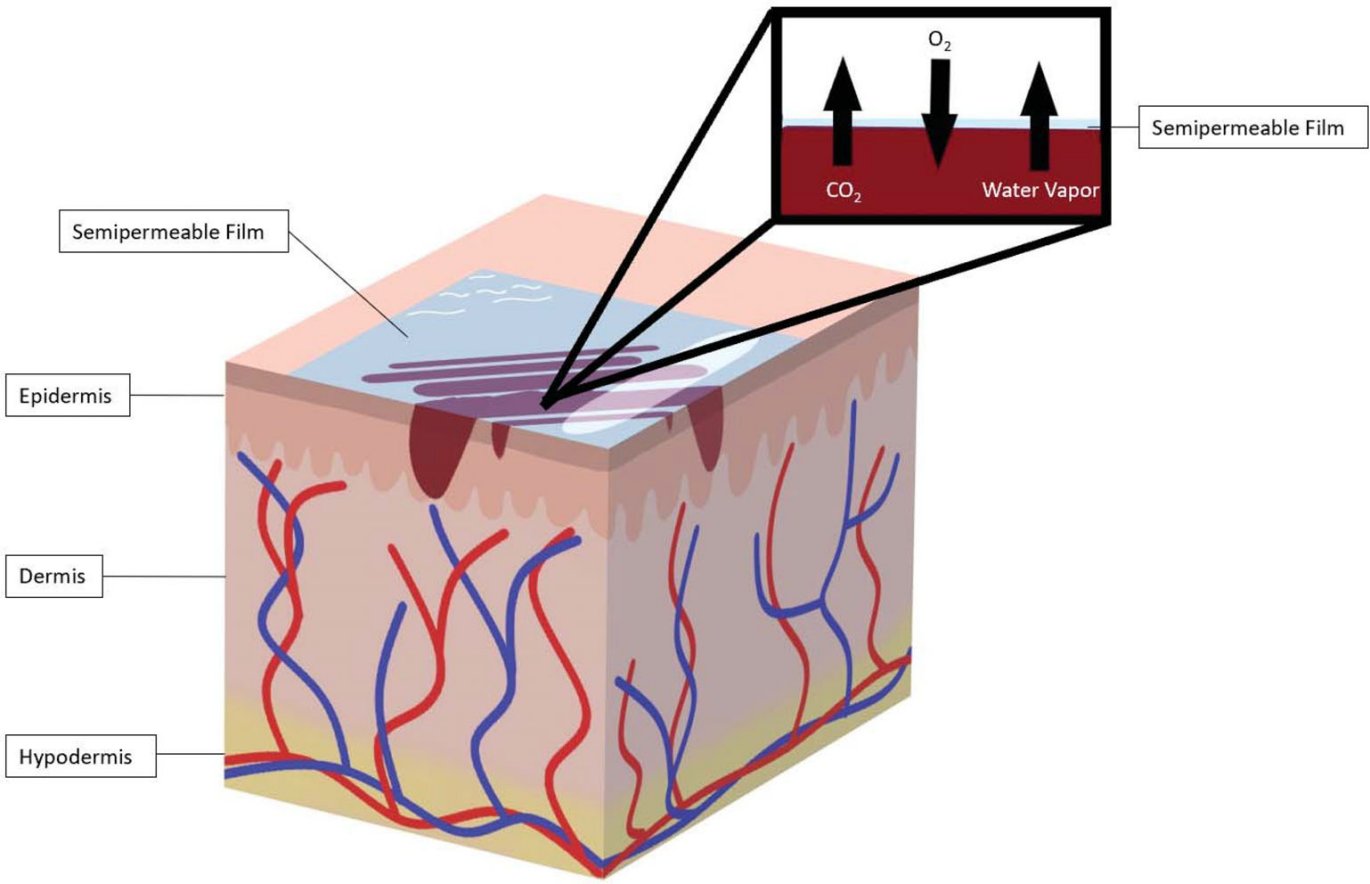
Semipermeable films. Semipermeable films are transparent, fluid impermeable wound dressings that allow the exchange of O_2_, CO_2_, and water vapor between the wound and the environment

Semipermeable films (Figure 2) are transparent films that are permeable to gases and impermeable to liquids and bacteria.^27^ The transparency of the films permits visualization of the wound, without requiring removal of the dressing.^27^ Semipermeable films allow enough oxygen to penetrate and affect growth of anaerobic bacteria, but not enough to support the underlying tissue growth.^24^ The waterproof nature of the films trap the bodily fluids in the wound, supporting autolytic debridement.^24^ Though semipermeable films support autolytic debridement, the impermeability to liquids can lead to a buildup of exudate which makes the surrounding wound tissue susceptible to maceration. The amount of water vapor escape through the film is influenced by the material of the film and the thickness of the material. It is measured as “mean vapor transmission rate” or MVTR. For example, just adding adhesive to the plastic film decreases the MVTR. A low MVTR can lead to the accumulation of exudate. So, this is an important parameter to know and understand in the use of films.

### 2.3 Hydrocolloids

**FIGURE 3:**
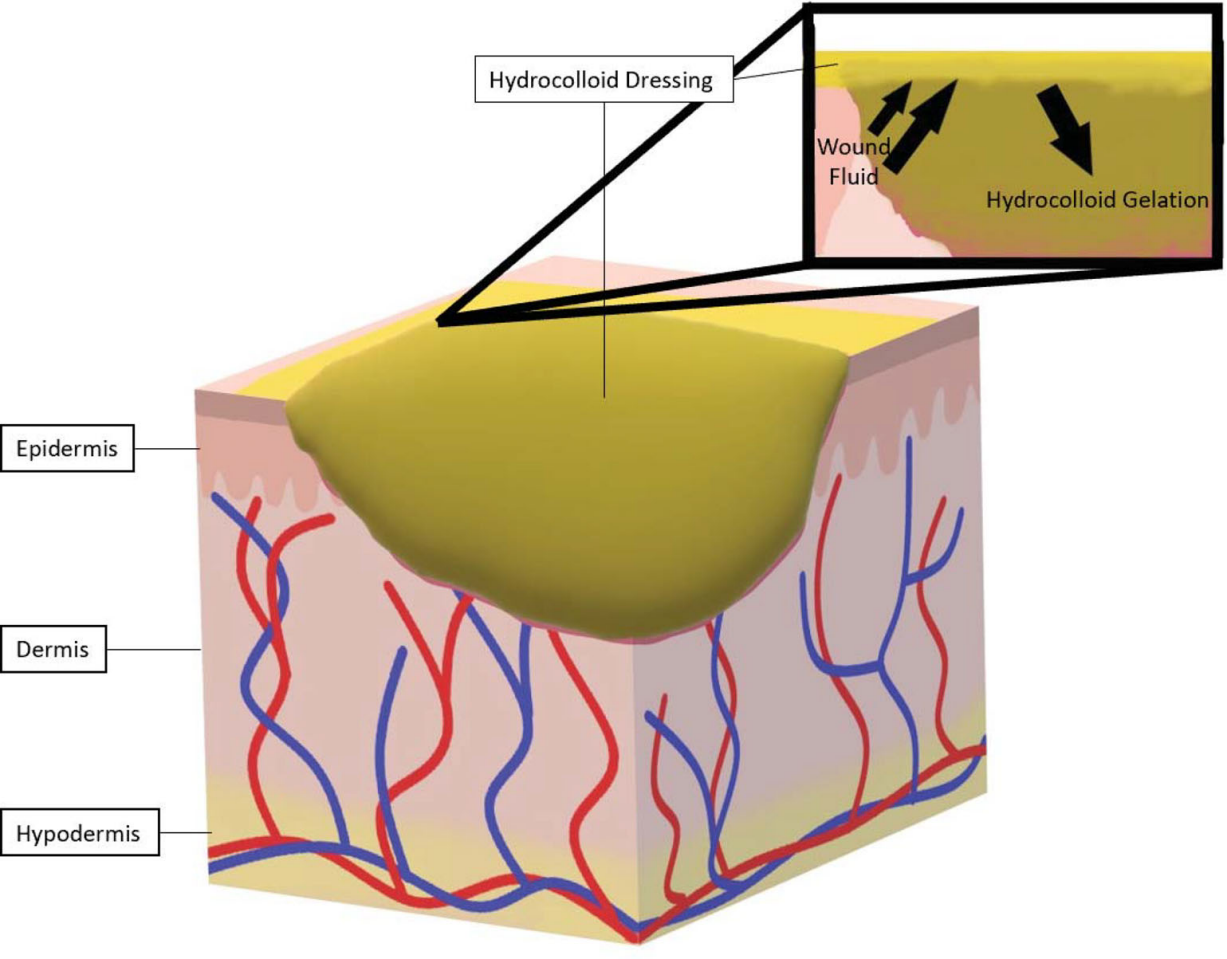
Hydrocolloids. Hydrocolloid dressings are usually opaque wound dressings that provide moisture to the wound by gelling on contact with wound fluid such as blood and exudate

Hydrocolloid wound dressings are usually composed of two layers: An outer semi‐occlusive layer and a hydrocolloid layer.^27^, ^34^ The outer occlusive layer is usually a foam or film. The hydrocolloid layer consists of a crosslinked polymer matrix that, in the presence of exudate, absorbs fluid to form a gel.^27^ This reaction results in a moist environment for the wound bed and stimulates autolytic debridement.^27^ Evidence suggests that hydrocolloid wound dressings are more effective for complete wound healing of chronic wounds than saline or paraffin gauze.^35^ Hydrocolloid dressings are usually opaque, which complicates wound inspection.^36^ Upon contact with wound exudate, hydrocolloids form a thick, yellow malodorous gel that can be mistaken for infection upon removal of the dressing.^36^ Though the gel both looks and smells unpleasant, it provides a cushioning effect and prevents the dressing from sticking to the wound, facilitating painless removal.^37^ Patients should be educated that this is a normal effect believed to be due to the breakdown products of gelatin.^38^ Clinicians should use other parameters such as warmth and erythema to assess for wound infection when using hydrocolloid wound dressings.^38^ See Figure 3 for an illustration of a hydrocolloid dressing.

### 2.4 Hydrogels

**FIGURE 4:**
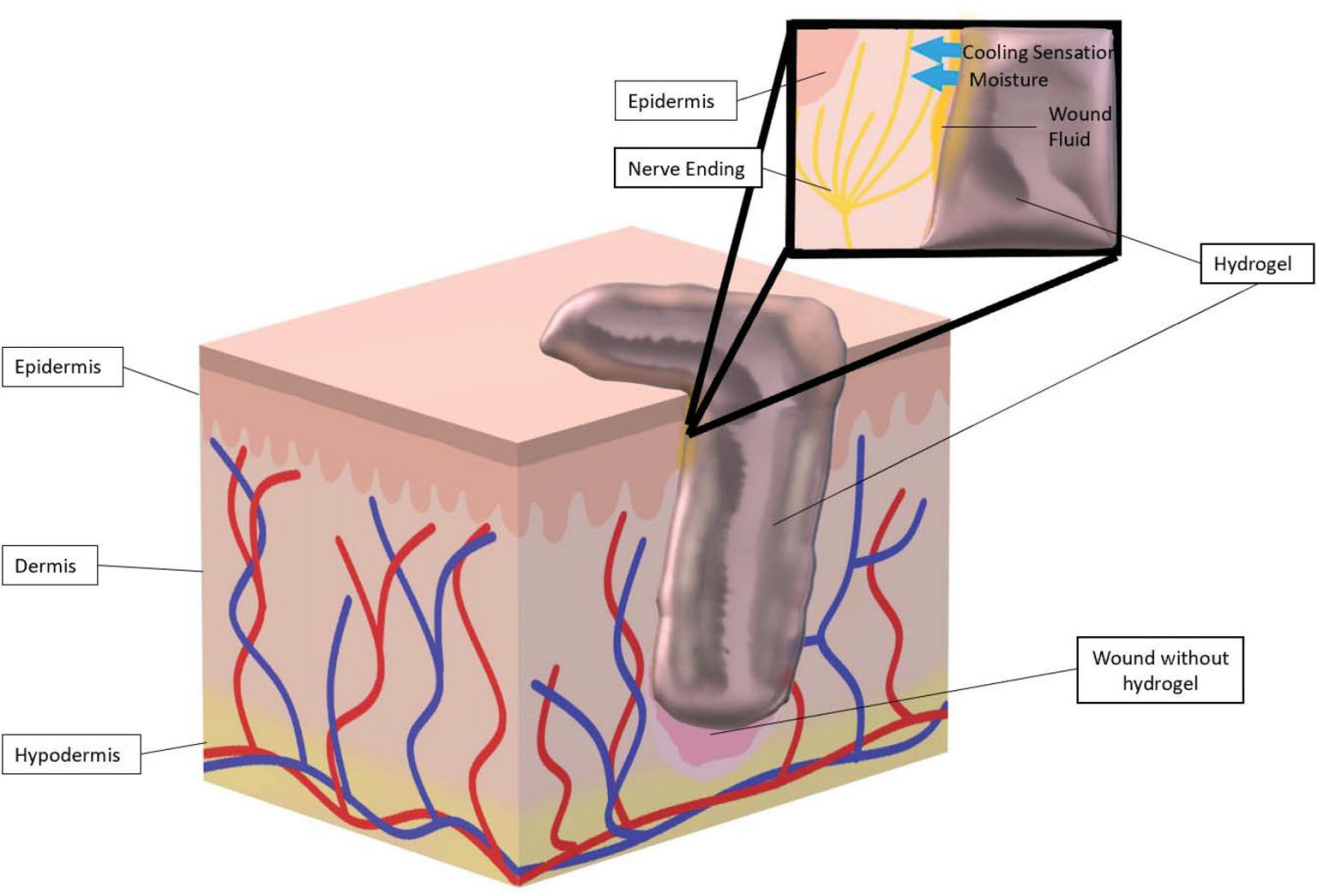
Hydrogels. A transparent amorphous hydrogel filling a cavitary wound is illustrated above. Hydrogels provide moisture to a wound and deliver soothing relief with via a cooling sensation

Hydrogel wound dressings consist of insoluble polymers in matrix with up to a 96% water content.^36^ Hydrogels are available in a multitude of physical forms: amorphous (Figure 4), sheets, and impregnated within gauze,^36^ nanoparticles, films, and coatings.^39^ Hydrogel properties can be manipulated by a developer's choice of polymeric materials and the method of crosslinking.^40^ They are also being explored as drug delivery systems.^39^ For example, Yan et al^41^ compared the effects treating deep partial thickness burns with a hydrogel containing recombinant granulocyte‐macrophage colony‐stimulating factor (GM‐CSF) to a hydrogel without additives and found that the hydrogel containing GM‐CSF had a faster healing time than hydrogel alone. Researchers at Xi'an Jiaotong University have actively been engineering hydrogels capable of self‐healing, hemostasis, antibacterial, and antioxidant capabilities.^42^, ^43^, ^44^, ^45^

Hydrogels maintain a moist environment in the wound bed and even provide a cooling effect when applied, which can act to soothe and reduce inflammation.^28^, ^36^ Hydrogels tend to have a low absorptive capacity due to its inherent high water content.^28^ Low absorptive capacity can facilitate exudate accumulation, which can lead to wound maceration and bacterial proliferation.^34^ They have low adherence to the wound bed, which necessitates use of a secondary dressing such as film or foam.^36^ Hydrogels have small pore sizes which can impede cell migration and the diffusion of proteins, waste products, nutrients, and oxygen.^38^ Cryogels are able to overcome the limitations imposed by the nanoporous hydrogel.

Cryogels are a subtype of hydrogel that are formed at sub‐zero temperatures, resulting in a macroporous structure.^38^ Cryogels have been used in various applications, such as tissue engineering, cosmetics, cell transplantation, and immunotherapy.^38^ Cryogels are able to be compressed at over 90% strain and regain their original configuration.^46^ The macroporous nature and injectability of the cryogels make them excellent candidates for hemostatic application.^47^, ^48^, ^49^

### 2.5 Foam

**FIGURE 5:**
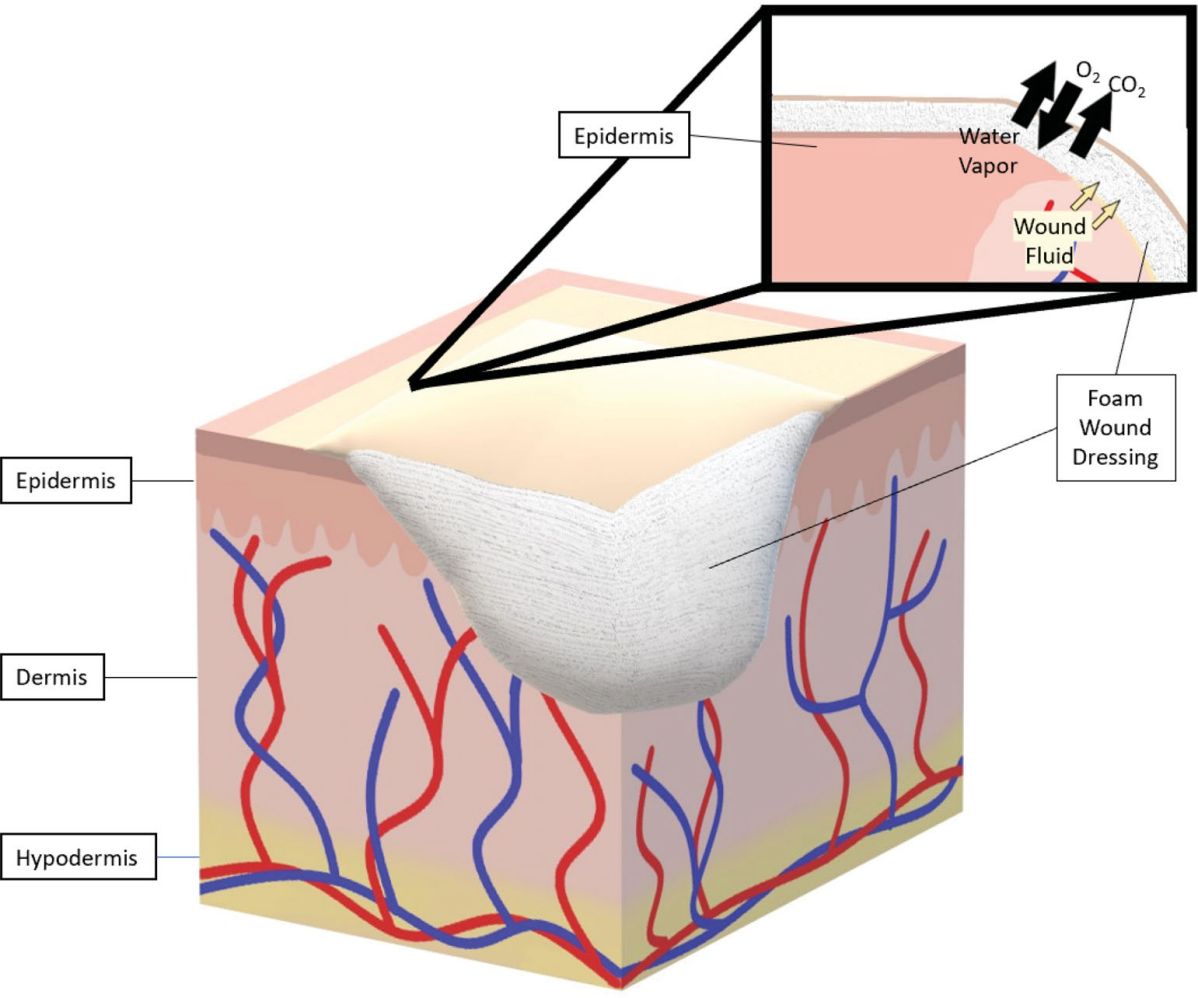
Foams. Foam wound dressings absorb blood and exudate, while allowing for the exchange of water vapor and gas between the environment and the wound bed

Foam wound dressings (Figure 5) are absorbent dressings that come in sheets and cavity filling chips.^50^ Foam's ability to absorb large amounts of exudate is dependent upon the composition and vapor transmission rate of the foam.^51^ Foam dressings provide protection from trauma and thermal insulation to the wound.^26^ Reticulated foam dressings can be combined with negative pressure wound therapy (NPWT) to remove drainage and exudate and further debride the wound.^50^ These open‐cell foam dressings are generally of a material that is hydrophobic so without the vacuum associated with NPWT they will not absorb or remove any fluid. Also, because they are reticulated and open‐celled, tissue ingrowth is rapid, and this prevents the migration of epithelium beneath the foam so this foam cannot be used on intact skin and cannot be used for epithelialization. Many available foam dressings are of hydrophilic material or configuration. They absorb fluid without the need for vacuum drainage. These foam materials can have the contact surface compressed so that the pore size, just like fine mesh gauze, does not admit any tissue ingrowth and these particular foams promote epithelial migration. In fact some of the fastest rates of epithelial migration reported by G. D. Winter were with these materials as dressings (e.g., Lyofoam).^52^, ^53^ It is recommended that highly absorbent foam dressings be avoided in patients with little to no exudate due to the risk of drying.^28^

### 2.6 Antimicrobial dressings

Antimicrobial agents can be added to wound dressings to prevent wound contamination. To prevent microbial wound invasion, wound dressings have incorporated substances, such as antibiotics, nanoparticles, and natural products.^54^ Simões et al^54^ excellently summarized recent investigations of antimicrobial wound dressings in a review. Table 3 provides a list of these antimicrobial agents and respective mechanisms of action that have been used in wound dressings.

**TABLE 3:** Antimicrobials found in wound dressings

Antimicrobial	Mechanism	Examples of pathogen coverage
*Antibiotics*		
β‐Lactams	Prevents synthesis of bacterial cell wall^174^	*Streptococcus pneumoniae* ^*175*^ *Listeria monocytogenes* ^*175*^ *Escherichia choli* ^*175*^ *Haemophilus influenzae* ^*175*^
Tetracycline	Binds to 30S ribosomal subunit to prevent protein synthesis^174^	*Mycoplasma pneumoniae* ^*176*^ *Chlamydiae trachomatis* ^*176*^ *Rickettsia rickettsii* ^*176*^ *Borrelia burgdorferi* ^*176*^
Aminoglycosides	Inhibition of protein synthesis^174^	*Escherichia coli* *Klebsiella pneumoniae* *Yersinia pestis* *Staphylococcus aureus*
Quinolones	Inhibition of DNA gyrase and topoisomerase IV^174^	*Salmonella enterica* ^*177*^ *Shigella sonnei* ^*177*^ *Escherichia coli* ^*177*^ *Pseudomonas aeruginosa* ^*177*^
Sulphonamides	Competitively inhibit dihydropteroate synthetase in folic acid synthesis pathway^174^	*Nocardia brasiliensis* ^*177*^ *Toxoplasma gondii* ^*177*^
Glycopeptides	Prevention of transglycosylation step in cell wall synthesis^174^	*Staphylococcus aureus* ^*178*^ *Streptococcus pnumoniae* ^*178*^
*Nanoparticles*		
Iron oxide	Disruption of DNA and enzymatic processes	
Titanium dioxide	Disruption of cell wall, plasma membrane, and DNA	
Zinc oxide	Disruption of cell wall and plasma membrane	
Silver	Disruption of cell wall and plasma membrane, DNA replication, transcription, and enzymatic pathways	*Neisseria gonorrhoeae* ^*179*^
*Natural products*		
Honey	Provides unfavorable environment for microbes, prevents microbial growth, damages cell walls, lipids, proteins, and nucleic acids	*Staphylococcus aureus* ^*180*^
Henna	Contains quinones which form stable free radicals that irreversibly complex with amino acids to inactivate proteins^181^	
Curcumin	Inhibition of cell division^182^	
Aloe vera	Contains anthraquinones, which is a structural analogue of tetracycline (mechanism of action listed above)^183^	
Thymol	Disrupts cell membranes^184^	
Essential oils: Cinnamaldehyde Geraniol Thymol analogues Menthol Carvacrol	Disrupts cell membranes	

### 2.7 Wound dressings by injury

There is currently insufficient evidence to draw conclusions about the best use of specific wound dressings in treating burns.^55^ The dressings that are typically used for Stage 1 and 2 pressure ulcers are polyurethane films, hydrocolloid wafers, and foam dressings.^56^ For full thickness ulcers (Stage 3 and 4 pressure ulcers) that are highly exudative, absorptive dressings, such as those consisting of calcium alginate, and gauze packing are recommended.^56^ Table 4 provides a quick reference for suitable wound dressings based upon wound types.

**TABLE 4:** Preferred wound types of various wound dressings^26^

Wound type	Low exudate	Medium exudate	High exudate
Flat, shallow wounds	Semipermeable Film Contact layer dressing Hydrocolloid Hydrogel	Semipermeable film Hydrocolloid Foam^26,28^ Alginates	Foam^26,28^ Alginates
Cavity	Hydrocolloid Hydrogel	Hydrocolloid Foam Alginates	Foam Alginates
Infected wounds	Antimicrobial	Antimicrobial	Antimicrobial

### 2.8 Other wound management techniques

**FIGURE 6:**
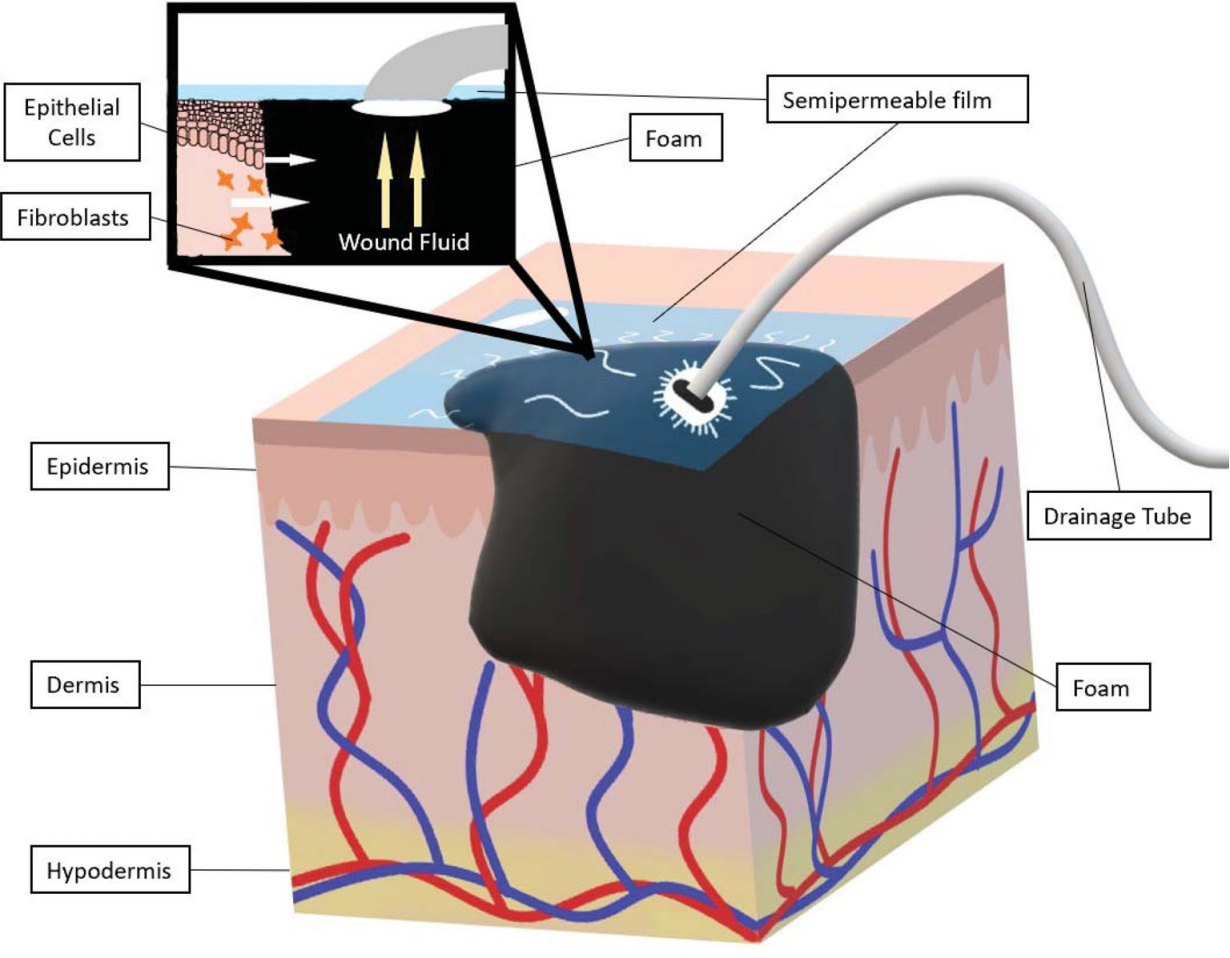
Negative pressure wound therapy. Negative pressure wound therapy involves the creation of a sub‐atmospheric environment in the wound, which enables wound edges to come together, facilitates fluid drainage, and stimulates fibroblasts and epithelial cell activity. The vacuum device is not pictured

NPWT is a system that utilizes subatmospheric pressure to enhance wound healing. NPWT devices consist of a semipermeable film covering a wound filler, most commonly polyurethane foam though gauze and polyvinyl foam can also be used, which either directly contacts the wound bed or rests on top of a low adherent contact layer.^57^, ^58^ The dressing is connected to a drainage tube which is also connected to a device responsible for generating the negative pressure.^58^ Figure 6 demonstrates an example of a NPWT system. NPWT pulls wound edges together, removes edematous fluid, stimulates fibroblast proliferation, and, with the use of a liner or contact layer, stimulates epithelial cell proliferation.^57^ Some contraindications of NPWT include malignancy, osteomyelitis, and the presence of necrotic tissue.^59^

Another wound management technique is shock wave therapy, which is hypothesized to utilize external deformations to the cytoskeleton via mechanotransduction.^60^ Shock wave therapy involves the absorption of biphasic high energy acoustic waves by tissue through electrohydraulics.^60^ Larking et al was able to demonstrate the effectiveness of extracorporeal shock wave therapy (ESWT) on chronic decubitus ulceration through a cross‐over study.^61^ Both study groups demonstrated wound healing after the introduction of ESWT.^61^ Current use of this wound management technique is limited due to a lack studies with high level evidence and a lack of optimization of shock wave parameters.^60^

Hyperbaric oxygen is being investigated as a treatment modality to promote wound healing. Hyperbaric oxygen therapy (HBOT) involves patients breathing 100% oxygen while being exposed to increased atmospheric pressure.^62^ One of the proposed mechanisms for this treatment modality's efficacy is through stimulation of neovascularization by increasing VEGF expression.^62^ A cross‐sectional study conducted in Brazil found venous ulcers, traumatic injuries, and diabetic foot wounds as the most common indications for HBOT.^63^ A systematic review including 29 studies on the effects of HBOT on skin wound healing found that 18 of those studies showed at least one positive outcome including wound closure and vascular perfusion.^64^ However, the studies included varying levels of evidence; five of the studies were randomized control trials with small sample sizes, five were prospective cohort studies, eighteen were clinical studies, and three were case series.^64^

Photobiomodulation (PBM) is a form of light therapy that uses nonionizing light sources to elicit photochemical and photophysical biological responses through absorption by chromophores.^65^ PBM is known to decrease pain and inflammation as well as promote wound healing and tissue regeneration.^66^ The exact mechanisms leading to these effects are still under investigation, though studies have shown that absorption of specific wavelength of light are absorbed by cytochrome C oxidase, initiating a cascade that results in generation of increased adenosine triphosphate and reactive oxygen species.^65^ PBM also results in activation of TGF‐β1 as well as the modulation of various ions, such as calcium, proton, sodium, and potassium between the cytosol and extracellular matrix.^65^

Ultrasonic debridement is a method of wound bed preparation used in the UK, Europe, and Australia.^67^ With ultrasonic debridement, saline runs through a tube that is attached to a headpiece; this headpiece vibrates at ultrasound frequency, creating bubbles which subsequently implode, producing a shockwave that is transmitted from the saline to the wound bed.^67^ The resulting shockwaves destroy dead and dying tissues while stimulating the more elastic membranes of healthy cells.^67^, ^68^ Ultrasonic debridement has been shown to promote angiogenesis, increased collagen deposition, mast cell degranulation, and increased intracellular calcium.^68^

## 3 BIOMATERIALS

### 3.1 Introduction to synthetic biomaterials

The biomaterials that are used to make wound dressings can be classified as either synthetic or natural. Synthetic biomaterials usually have good mechanical properties, thermal stability, and a shape that can be manipulated.^69^ Synthetic biomaterials can be further classified into metals, polymers, ceramics, and composites.^70^ Polymeric and composite materials will be considered below.

### 3.2 Polyurethane

Polyurethane is a synthetic polymer consisting of repetitive urethane groups that is, used as a biomaterial in both semipermeable films and foam wound dressings.^71^ It is synthesized through polymerization of diisocyanates and polyols.^72^ Properties of the polyurethane may vary based upon the choice of diisocyanate, polyol, and chain extender used in its synthesis.^72^ For example, Yoo et al^73^ synthesized polyethylene glycol‐based waterborne polyurethane hydrogels with desirable water absorption percentage and water vapor transmission rates. Polyurethane is viewed as a desirable biomaterial for wound dressings due to its biocompatibility, strength, and lack of toxicity.^71^

### 3.3 Silicone

Silicone is a biomaterial used in foam dressings and low adherent contact wound dressings, such as Adaptic™ and Mepitel®.^74^ It is a polymer of repeating units of siloxane, which is composed of silicon, oxygen, and an alkane.^75^ Most medical products that contain silicone utilize the organic polymer polydimethylsiloxane.^75^ Silicone is nontoxic, biocompatible, and resistant to biodegradation in the short term.^70^ A review by Truong et al found that silicone foam dressings were more effective than traditional methods, such as regular repositioning and skin care, at preventing pressure ulcers.^76^

### 3.4 Carboxymethylcellulose

Carboxymethylcellulose is a derivative of cellulose that contains a carboxymethyl group bound to the hydroxyl groups found in the backbone of the cellulose molecule.^70^ It is synthesized in a two‐step reaction that consists of the alkalization of cellulose, followed by carboxymethylation using chloroacetic acid.^77^ Carboxymethylcellulose is a nontoxic, nonimmunogenic, and biocompatible polymer.^78^ It is hydrophilic and rapidly undergoes gelation in an aqueous environment.^79^ Examples of wound dressings that carboxymethylcellulose is used for include hydrocolloids, hydrogels, and hydrofibers. DuoDERM® is a hydrocolloid wound dressings offered by the company ConvaTec Inc that contains a proprietary mix of carboxymethylcellulose, gelatin, and pectin.

### 3.5 Poly (vinyl alcohol)

Poly (vinyl alcohol) is a synthetic polymer synthesized from vinyl acetate by controlled free radical polymerization.^80^ Subsequently, the acetate group is removed from the resultant poly (vinyl acetate) via ester hydrolysis.^80^ The degree of hydrolysis, tacticity, and average molecular weight of the poly (vinyl alcohol) determine its solubility, biodegradability, and mechanical strength.^81^ Poly (vinyl alcohol) is biocompatible, easily prepared, odorless, and has a low permeability to oxygen and aroma.^81^ Due to poly (vinyl alcohol) having poor stability in water, inadequate elasticity, and a rigid structure,^81^ it is usually blended with synthetic polymers, such as polyvinylpyrrolidone, polyethylene glycol, poly (*N*‐isopropylacrylamide), and montmorillonite, or natural polymers, such as chitosan, alginate, starch, dextran, glucan, gelatin, and hyaluronan. An example of such a dressing would be Hydrofera Blue®, which is composed of polyurethane and poly (vinyl alcohol).^82^

### 3.6 Poly (ethylene glycol)

Poly (ethylene glycol), also referred to as poly(ethylene oxide) when the molecule weight exceeds 20,000 is a neutral polyether used in for a wide range of biomedical and biotechnical applications.^83^ Poly (ethylene glycol) is a nonimmunogenic and biocompatible hydrophilic polymer.^84^ Poly (ethylene glycol) is soluble in water and many organic solvents, but becomes insoluble in water when exposed to temperatures above 100°C.^83^ Though poly (ethylene glycol) is bioinert in nature, it can be modified to incorporated bioactive moieties.^85^, ^86^ Poly (ethylene glycol) can be functionalized by either replacing the terminal hydroxyl groups with a functional group or by reacting the poly (ethylene glycol) with a molecule that has two functional groups, one of which will be replaced with poly (ethylene glycol).^87^ Poly (ethylene glycol) modifies both the solubility and size of a molecule to which it is attached.^87^

### 3.7 Composites

Composite wound dressings consist of two or more distinct elements. A biocomposite material consists of two or more matrices.^88^ Biocomposite materials have improved properties compared to the individual components. For example, Park et al^89^ synthesized a composite wound dressing consisting of chitosan and silica that was shown to lead to an increase in collagen deposition compared to a chitosan sponge alone. One of the first “composite” dressings proposed as such (1981) was by a group of scientists at 3M that included G.D. Winter posthumously.^25^ This was a combination of polytetrafluoroethylene fibril matrix, hydrophilic absorptive particles enmeshed in the matrix and a semi‐occlusive film on the outer surface. This has become a model for many subsequent dressing products.

### 3.8 Introduction to natural biomaterials

Biomaterials that are derived from animals, plants, and microbes are considered to be natural.^70^ Natural biomaterials are typically biocompatible, biodegradable, and possess similar properties to the extracellular matrices found in connective tissues.^90^ In addition, natural biomaterials are advantageous because of their ability to interact at the molecular level with tissues, thereby playing an active role in the wound healing process.^70^

### 3.9 Collagen

Collagen is the most abundant protein found in mammals, comprising a third of one's total protein content.^91^ The collagen polypeptide is rich in proline, hydroxyproline, and glycine that displays a characteristic triple helix conformation.^92^ The enzyme lysyl oxidase crosslinks the lysine and hydroxylysine residues, providing strength and stability to mature collagen fibrils.^92^ Collagen affects all phases of wound healing; collagen acts as a matrix for blood clot formation thereby promoting hemostasis, scavenges reactive oxygen and reactive nitrogen species, acts as a sacrificial substrate for collagenases, and promotes migration of fibroblasts, macrophages, and epithelial cells into the wound area.^93^ Collagen wound dressings are flexible, nontoxic, biodegradable, and have low immunogenicity.^93^ Collagen wound dressings are absorbent and can decrease the pH of wound fluid, decreasing the likelihood of bacterial colonization as well as the activity of proteases.^93^ Collagen wound dressings are recommended for full and partial thickness wounds with little‐to‐moderate exudate.^70^ Collagen wound dressings are available in various forms, such as powder (e.g., Stimulen™) that dissolves in wound fluid to form a gel, matrices (e.g., Biostep™ by Smith & Nephew), and freeze dried composites (e.g., Promogran Prisma).

### 3.10 Alginate

Alginate is derived from brown seaweed in the Phaeophyceae class.^94^ It is composed of linear copolymers of (1,4)‐linked β‐D‐mannuronate and α‐L‐guluronate blocks.^94^ Alginate forms gels in the presence of acid and divalent and trivalent cations through an ionotropic mechanism.^95^ In the case of sodium alginate gelation, sodium alginate exchanges its sodium cation that was bonded to guluronate with the divalent or trivalent cations, which link the polymers together.^94^ The proportion of mannuronic and guluronic acid determines alginate's physical and chemical properties; a greater mannuronate content promotes gelation while a greater guluronate content promotes fiber integrity.^96^ Alginates can be used to form many different types of wound dressings including hydrogels, semipermeable films, foams, and nanofibers.^95^ According to a chapter in *Advanced Wound Repair Therapies* written by Agarwal et al,^96^ the benefits of using alginate based wound dressing include gelation providing a moist wound healing environment, ability to absorb 20–30 times its weight in exudate, hemostatic capabilities, modulation of wound macrophages, and, reportedly, enhanced epithelialization and granulation tissue formation. Antimicrobials, medications, and nanoparticles can be added to alginate based wound dressings to enhance its antimicrobial activity. It is recommended that alginates be avoided in wounds that contain little exudate to no exudate and changed often due to the possibility of desiccation of the dressing. This can cause adherence of the dressing to the wound, which would result in painful removal.^24^, ^26^, ^36^ One example of an alginate wound dressing is the Kaltostat® calcium sodium alginate dressing sold by ConvaTec.

### 3.11 Chitin and chitosan

Chitin is the second most abundant natural polysaccharide, and consists of polymers of β (1, 4) linked *N*‐acetylglucosamine units.^97^ The main source of chitin used for commercial purposes are the shells of crustaceans, though it can also be found in the exoskeletons of other arthropods as well as in the cell walls of fungi and yeast.^98^ Chitosan can be synthesized by partial deacetylation of chitin.^98^ Chitin and chitosan are both biocompatible, biodegradable, nontoxic, and nonimmunogenic.^97^ When chitin and chitosan are degraded by lysozymal activity they form *N*‐acetyl‐β‐D‐glucosamine which has been found to stimulate (a) fibroblast proliferation, (b) ordered collagen deposition, and (c) hyaluronic acid synthesis.^97^ Both chitosan and chitin can be used as a biomaterial for various types of wound dressings, including hydrogels, sponges, films, and porous membranes.^99^ A couple of chitosan product examples include the HemCon® Bandage Pro, Smith & Nephew BST‐CarGel®.

### 3.12 Pectin

Pectin is a heteropolysaccharide consisting of several different structural elements, such as homogalacturonan and rhamnogalacturonan, that can be found in the cell walls of dicotyledons.^91^ Pectins that have greater than 50% methylation in the homogalacturonan region will gel in an acidic environment (pH <4.0) in the presence of a high concentration of sugar while pectins with a lower degree of methylation gel in the presence of divalent cations regardless of the presence of soluble solids.^91^ Pectin is useful in biomedical applications due to its biocompatibility, biodegradability, low cost of production, and availability.^100^ Pectin has been used as a biomaterial for hydrocolloids,^91^ hydrogels, and films.^100^ Two examples of wound dressings that contain pectin are ConvaTect Ltd.'s DuoDERM® and GranuGEL®.^101^

### 3.13 Hyaluronan

Hyaluronan (also known as hyaluronic acid) is a polysaccharide consisting of D‐glucuronic acid and *N*‐acetyl‐D‐glucosamine disaccharide.^102^ Hyaluronan is highly absorbent; when hydrated, it forms stiff helices that can contain as much as 1,000‐fold more water than polymer.^103^ Hyaluronan is naturally found in the extracellular matrix, vitreous humor, cartilage, synovial fluid, epidermis, and dermis.^91^ It is able to stimulate wound healing through multiple mechanisms. Hyaluronan modulates the inflammatory process, which may contribute to granulation tissue stabilization.^104^ It is able to soften the fibrin clot, facilitating cell colonization.^104^ In addition, hyaluronan modulates angiogenesis and stimulates fibroblasts.^104^

### 3.14 Gelatin

Gelatin is a protein derived from type I collagen present in bones and skin.^91^ Gelatin contains an amino acid sequence similar to arginylglycylaspartic acid, which is known to promote cell adhesion and migration.^105^ Gelatin forms a thermoreversible gel; it gels at lower temperatures and melts when the temperature increases.^106^ At physiological temperatures, gelatin gel is unstable unless it is chemically or enzymatically crosslinked. Gelatin has been used in hydrocolloids, hydrogels, and foams. Two examples of gelatin based sponges are Surgifoam® Absorbable Gelatin Sponge (Johnson & Johnson) and Gelfoam® (Pfizer). Gelatin is also present in some hydrocolloid dressings, such as Granuflex®,^91^ and the aforementioned DuoDERM.

## 4 DISCIPLINES OF WOUND DRESSING DEVELOPERS

**FIGURE 7:**
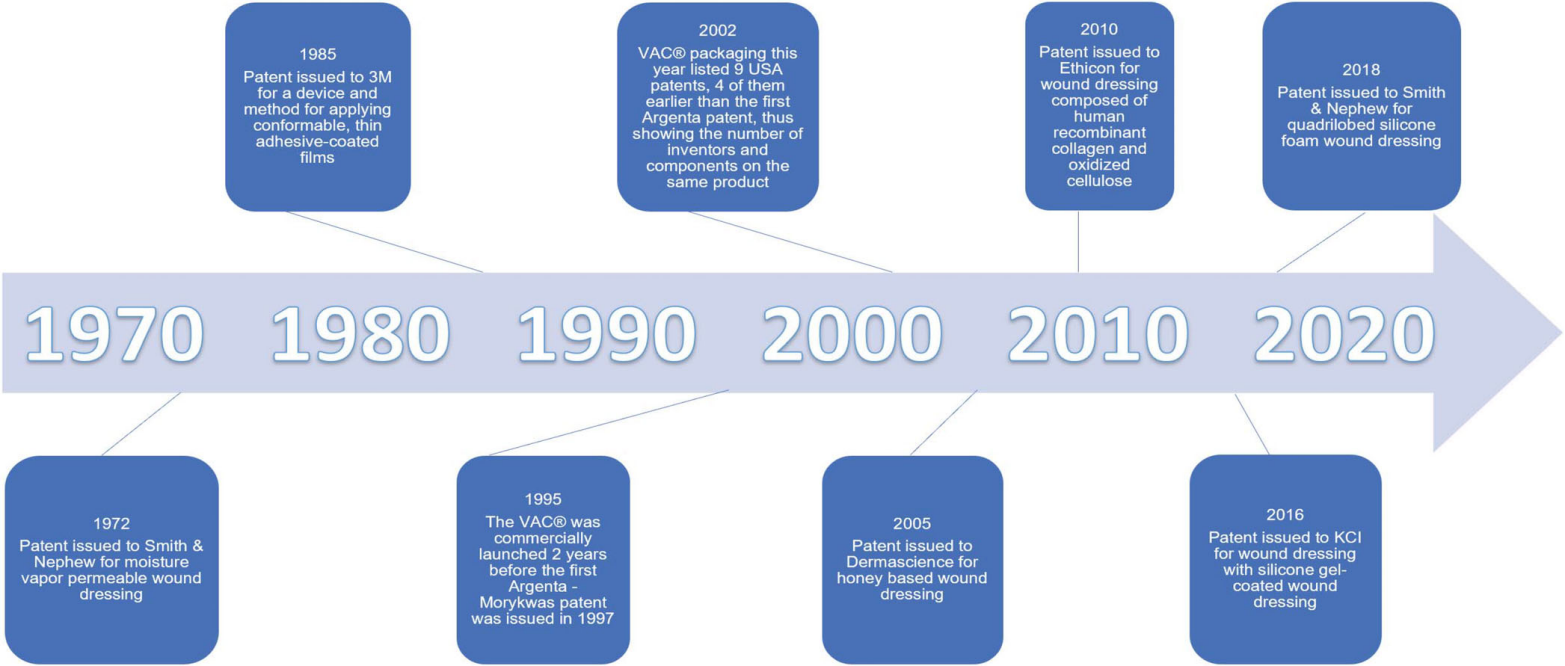
A timeline of the issuance of patents for various wound healing technology companies

According to a paper published in 2017 by Gwak et al,^107^ the top five applicants for patents for wound healing technologies with the United States Patent and Trademark Office (USPTO) include Kinetic Concepts, Inc (KCI), Smith & Nephew, 3M, Johnson & Johnson, and Tyco Healthcare. With the recent (October 2019) purchase of KCI by 3M, this puts 3M in an even stronger position in this list. This section will describe wound dressings assigned to the aforementioned top companies submitting patents, wound dressings sold by a relatively new company, and the respective inventor backgrounds. Tyco Healthcare will not be considered below due to the fact it was spun off by Tyco International in 2007. See Figure 7 for a timeline of the events described below.

### 4.1 Johnson & Johnson

According to its company website, Johnson & Johnson was founded in 1886 by three brothers: James Wood Johnson, Robert Johnson, and Edward Johnson. Johnson & Johnson offers many products addressing different aspects of a wound healing including Instat® and Surgicel®. Instat is a collagen sponge sold by Johnson & Johnson indicated for hemostasis. According to its trademark history, Instat was first used commercially in April of 1985. The first collagen sponge patent from Johnson & Johnson was published in 1962.^108^ The patent was assigned to Ethicon, Inc., a Johnson & Johnson subsidiary, and outlined a process for preparing a collagen sponge that was water insoluble and able to retain its original rigid structure. The inventor listed on this project is scientist Dr. Charles Artandi, Ph.D. Dr. Charles Artandi was the former Associated Director of Research at Ethicon, Inc. He has multiple research publications, most relating to radiation and the use of radiation for sterilization.^109^, ^110^, ^111^, ^112^, ^113^, ^114^ The process of producing a collagen sponge was an improvement upon the method patented by scientist, Dr. Robert H Sifferd, and chemical engineer, Ralph J Schmitt.^115^ Dr. Sifferd has written research papers with biochemists involving amino acids and sterols.^116^, ^117^, ^118^, ^119^ The patent describes the use of surgical sponges to stop the flow of blood and fluid, and the use of collagen to provide a stronger sponge with a “highly desirable physical structure.”

Surgicel is an absorbable hemostat consisting of oxidized regenerated cellulose. According to the USPTO, the Surgicel trademark was first used in commerce in November of 1957. Wound dressings containing cellulose; however, have been available for over a century. In 1901, chemical engineer Henry P Weidig filed an application with the USPTO for a surgical dressing consisting of cellulose and pyroxylin (a form of nitrocellulose).^120^ A year later, engineer Robert W. Johnson, better known as the co‐founder of Johnson & Johnson, submitted a patent application for an absorbent surgical dressing.^121^ This dressing consisted of absorbent layers of “rumpled cellulose tissue‐paper,” separated by a thin layer of absorbent cotton. In 1938, chemists Edward Yackel and Dr. William Kenyon file a patent, assigned to Eastman Kodak Co, describing a method of preparing oxidized cellulose.^122^ Eastman Kodak Co. supplied oxidized cellulosic gauze through Parke, Davis & Company for physicians to use for research.^123^ According to the USPTO, Parke, Davis & Company trademarked the absorbable cellulose dressing as Oxycel® in 1944. Many physicians published research using Oxycel soon after.^124^, ^125^, ^126^, ^127^, ^128^ Chemist and former head of the Johnson & Johnson research department David F. Smith filed two patents involving hemostats consisting of cellulose in 1950 and 1957.^129^, ^130^ Johnson & Johnson filed for the Surgicel trademark in 1958.

A patent was issued for a collagen and oxidized regenerated cellulose sponge in October of 2001 with Johnson & Johnson as the assignee. In April of 2002, Johnson & Johnson announced the release of Promogran™ Matrix Wound Dressing, a wound dressing comprised of oxidized regenerated cellulose, collagen, and silver. During that year, Dr. Breda Cullen, Ph.D., along with other scientists at Ethicon published research papers regarding the effects of oxidized regenerated cellulose and collagen on wound healing.^131^, ^132^, ^133^, ^134^ Physicians also begin to publish research using the Promogran Matrix Wound Dressing, some of which received a grant from Johnson & Johnson.^135^ In December of 2005, Dr. Breda Cullen and two other scientists from Ethicon filed an application for a wound dressing composed of human recombinant collagen and oxidized cellulose.^136^ The patent was issued by USPTO in November of 2010. Research involving the collaboration between the inventors and physicians was published many years after the initial filing of the patent regarding the use of oxidized regenerated cellulose and collagen as wound dressings.^137^, ^138^, ^139^, ^140^ The wound dressings that have the trademark Promogran are now sold by KCI.

### 4.2 Kinetic Concepts, Inc

KCI was an Acelity Company that, according to a 3M news release, was be acquired by 3M in October of 2019. In 1995, KCI trademarked Vacuum Assisted Closure, a brand of NPWT that, according to the KCI website, has more published clinical evidence than any other NPWT system. In 1991, Dr. Louis Argenta, a plastic surgeon, and Michael Morykwas, a biomedical engineer, from Wake Forest School of Medicine filed a patent with the USPTO for the NPWT system that would become Vacuum Assisted Closure®.^141^ However, at the time of commercial launch of the product in the early 1990's, Wake Forest had still not received an issued patent for their format for NPWT. KCI purchased patents from another physician and used patents from its own development engineers to launch the product under patent protection. The package label from 2002 for the product shows 15 patent labels; this demonstrates the work of multiple “inventors” necessary to develop the components for a complex device or product. Here again we see the combined work of practicing clinicians and company engineers represented in the patent literature. But for commercial success, there is no substitute for a clinical champion, and KCI had that in the Wake Forest pair of Drs. Argenta and Morykwas.^142^, ^143^

Another product offered by KCI, trademarked as Adaptic Touch™, is a low adherent contact layer dressing. Adaptic Touch consists of a cellulose acetate gauze coated with soft tack silicone. A USPTO patent, currently assigned to KCI, for a silicone gel‐coated wound dressing was filed by scientists in 2011, with a United Kingdom patent filed in 2010 taking priority.^144^ The patent for this invention was issued in March of 2016. A divisional patent, also assigned to KCI, for a wound dressing with a tacky silicone coating was filed by scientists in 2016.^145^ The patent was recently issued by USPTO in June of 2019. This patent cites many resources, including research publications primarily written by physicians.

### 4.3 3M

One of the patents cited was filed in 1981 for a polytetrafluoroethylene wound dressing coated with a silicone film, designed to prevent wound desiccation.^25^ It was issued by the USPTO in February of 1983. The patent was assigned to 3M and lists the inventors as Louis A. Errede, James D. Stoescz, and George D. Winter, all of whom are scientists. To date, 3M sells multiple styles of silicone foam wound dressings and the patent has been cited over 500 times. There are experimental results reported in this patent application that are not searchable in any other publication. The “trade” that a patent applicant makes is to agree to the publication of the application in exchange for proprietary patent protection for a specified period of time. That is the difference between the patent literature and “trade secrets” which are preserved inside a company and not accessible at all outside that company.

### 4.4 Smith & Nephew

Smith & Nephew is a medical technology company founded in England in 1856 by Thomas James Smith. Smith & Nephew has many signature brands including OpSite™, Allevyn™ Life, and Durafiber®. Smith & Nephew offers a uniquely shaped silicone foam wound dressing under the trademark Allevyn Life. Allevyn Life comes in a quadrilobed shape, as well as shapes that contour to the heel and sacrum. In 2016, inventors Ella Mumby and Dr. Helene Anne Lecomte, Ph.D., filed a patent application for a wound dressing with the characteristic four lobed shape.^146^ The patent inventors have a chemistry background and listed Smith & Nephew as the assignee. The wound dressing described in the patent contains an adhesive layer, a perforated wound contact layer, a polyurethane foam layer, a layer of activated charcoal cloth, a superabsorbent layer with cellulose fibers and polyacrylate, a shielding layer (consisting of either foam, woven fabric, or nonwoven fabric), and a top film layer. This patent was issued by the USPTO in August of 2018.

### 4.5 Comparison of Smith & Nephew and 3M: Semipermeable film

Opsite is a polyurethane film wound dressing that was introduced in 1977. Five years earlier, two patents were published by scientists listing Smith & Nephew as the assignee for polyurethane film wound dressings.^147^, ^148^ In 1975, Smith & Nephew filed to trademark the name OpSite for the selectively permeable films. In between the time of publishing the patent and the introduction of OpSite, plastic surgeons published research on the product.^149^, ^150^, ^151^, ^152^, ^153^, ^154^

In 1978, 3M began working on its own semipermeable film. 3M filed an application with the Canadian Intellectual Patent Office in 1981 for a method and device for applying an adhesive coated polymeric film, listing Steven Heinecke as the inventor.^155^ Steven Heinecke was an engineer at 3M that described going to a local hospital and interacting with critical care nurses to discover a need for a transparent IV site dressing.^156^ That same year, 3M test launched a semipermeable polymeric film known as Tegaderm™ in the United States. Six years later, 3M became the US market share leader in transparent film wound dressings. In 2005, the Tegaderm brand was extended to other 3M wound dressings including hydrocolloids, alginates, silicone foam, and so forth. Tegaderm is also the brand of adhesive film packaged with the KCI products VAC® and Prevena™.

### 4.6 Integra LifeScience

Integra LifeScience is a company that, even though it was founded relatively recently in 1989, has become one of the world leaders in medical devices. Integra LifeScience moved to acquire the tissue regeneration company Dermasciences, Inc in 2017. In July of 2015, inventors Keith Johnson, Rehan Khanzada, Mario Neto, and Barry Wolfenson filed a patent application for a foam wound dressing impregnated with honey assigned to Dermasciences, Inc.^157^ Two of the inventors have engineering backgrounds: One chemical, the other biomedical. Interestingly, all, except one inventor, have a background or education in business. To date, the patent status is listed as ready for examination. Integra LifeScience now offers *Leptospermum* honey‐based pastes, hydrogels, hydrocolloid wound dressings, and calcium alginate dressings under the trademark MEDIHONEY®. MEDIHONEY previously listed four issued patents on the Dermascience website.^158^, ^159^, ^160^, ^161^ One of the patents was filed by an inventor with a background in engineering.^160^ Two of the patents were filed by an inventor with a background in biochemistry.^158^, ^159^ The other patent was filed by an inventor with a background in farming and engineering.^161^ Multiple publications were cited in each of the patents, which were authored by primarily by biochemists, physicians, and chemists.^162^, ^163^, ^164^, ^165^, ^166^, ^167^, ^168^, ^169^

## 5 DISCUSSION

One question that should be explored is if early collaboration with different fields leads to a better wound dressing. A great example of this dynamic is seen with the product Tegaderm. It cannot be ignored that this product discovery was influenced by an engineer physically visiting a hospital and interacting with health care professionals that harbored a need. That engineer worked with a team of scientists to build a product that met those needs. Even though OpSite had been introduced around 6 years prior to Tegaderm's introduction to the US market, Tegaderm was able to surpass OpSite as a US market share leader in transparent films.

Another example of early collaboration leading to a better wound dressing is the advent of the Vacuum Assisted Closure device. This device not only introduced a new product, but it also introduced a new way of achieving wound healing, NPWT. Ten years after the original VAC® product was launched, work in a hospital setting by a plastic surgeon and an orthopedic surgeon working on difficult, infected joint replacements, produced a new modification of the original NPWT product and the patent was purchased by KCI.^170^ Working with the company engineers the Prevena™, an incisional dressing, was developed and launched. A meta‐analysis performed by Strugala et al^171^ demonstrated on the Prevena that a single use of NPWT resulted in the reduction of surgical site infection, wound dehiscence, and length of stay when used prophylactically.

Early collaboration between those in different fields is also seen with the development of the Medihoney products. The backgrounds of the Medihoney product inventors were diverse, including fields, such as business, farming, biochemistry, and engineering. One of the inventors even collaborated with physicians on research involving using honey as a dressing for leg ulcers and included the resulting publication on the patent application.^166^ Medihoney has since grown into the global leading medical honey‐based product line.

Early collaboration between professionals in different disciplines has been shown to be advantageous for innovations in wound management technologies. One possible reason could be that representatives of separate disciplines can bring different perspectives that can be brought together to solve a problem. Another possible reason is that early input from medical professionals that will be using the technologies or have firsthand experience of the problem that the product is designed to solve can help shape the development in a way that better addresses the issue experienced in clinical practice. In the future, deliberately utilizing interdisciplinary collaboration may prove beneficial when developing novel wound care products. There are untapped viewpoints and solutions that could enhance the creation of future medical technologies. Valuing diverse perspectives from differing fields of study and expertise can drive this innovation. Therefore, inventors should prioritize receiving early input from both scientists and clinicians when developing products intended for use in the delivery of health care.

There continues to be promising research that is likely to result in innovation in wound dressing technology. Current areas of active research in wound dressings include further exploring the use of chitosan as a biomaterial, experimenting with different biomaterials in the synthesis of hydrogels, utilizing electrospinning in the construction of the biomaterials, and incorporating a drug delivery system and nanoparticles in the matrix of wound dressings. For example, Long et al^172^ was able to 3D print a chitosan‐pectin hydrogel impregnated with the lidocaine hydrochloride that could release the anesthetic in a controlled manner over the course of hours in vitro. In this study, the release of lidocaine hydrochloride resulted in degradation of the hydrogel. This illustrates an ever‐present challenge that wound dressing pioneers are faced with, which is manipulating the delicate balance between structure and function in a way enhances the dressings wound healing capabilities.

## CONFLICT OF INTEREST

Ms. Hawthorne has no conflict of interest to disclose. Mr. Simmons has no conflict of interest to disclose. Mr. Stuart has no conflict of interest to disclose. Dr. Tung has no conflict of interest to disclose. Dr. Zamierowski reports relevant financial activity from KCI, Inc., outside the submitted work. In addition, Dr. Zamierowski has a patent 4,969,880 sold to KCI, and a patent 7,976,519 with royalties paid by KCI. Dr. Mellott reports relevant financial activity from Zam Research LLC and from Ronawk LLC, outside the submitted work. In addition, Dr. Mellott has a patent Expandable cell culture substrate (WO2018044990A1) licensed, and a patent Biopsy punch device and method (US201802349988A1) pending.

## Data Availability

Data sharing not applicable to this article as no datasets were generated or analyzed during the current study.
